# Mixed Milk Feeding Patterns and Growth Outcomes During the First Year of Life in Asian Infants: Application of Predefined Feeding Clusters to Test Associations

**DOI:** 10.1016/j.cdnut.2025.107565

**Published:** 2025-10-03

**Authors:** Liandre Frances van der Merwe, Kelly A Mulder, Floor M van Oudenhoven, Lynette P Shek, Oon Hoe Teoh, Wei Wei Pang

**Affiliations:** 1 Danone Research & Innovation, Utrecht, The Netherlands; 2 Department of Paediatrics, Yong Loo Lin School of Medicine, National University of Singapore, Singapore; 3 Department of Paediatrics, KK Women’s and Children’s Hospital, Singapore; 4 Global Centre for Asian Women’s Health, Yong Loo Lin School of Medicine, National University of Singapore, Singapore; 5 Department of Obstetrics & Gynaecology, Yong Loo Lin School of Medicine, National University of Singapore, Singapore; 6 Bia-Echo Asia Centre for Reproductive Longevity and Equality, Yong Loo Lin School of Medicine, National University of Singapore, Singapore; 7 Institute for Human Development and Potential (IHDP), Agency for Science, Technology and Research (A∗STAR), Singapore

**Keywords:** infant growth, mixed milk feeding patterns, multivariate k-means clustering, linear mixed-effects, WHO *z*-scores

## Abstract

**Background:**

Varying definitions have been used to categorize mixed milk feeding (MMF) patterns in studies focused on feeding and infant growth, posing a challenge when making comparisons and interpretations. Furthermore, MMF encompasses vastly heterogeneous and evolving feeding behaviors that are difficult to standardize longitudinally.

**Objectives:**

We previously described a new approach for describing MMF patterns across the first year, using a multivariate clustering algorithm. In the current article, we aimed to describe the associated infant growth patterns across these identified feeding clusters in an Asian clinical study (*N* = 539).

**Methods:**

Using a linear mixed-effects model that included infant covariates, we estimated the associations between the different feeding clusters and longitudinal growth outcomes, including weight, length and BMI-for-age *z*-scores.

**Results:**

The cluster division explained a statistically significant amount of variation in growth trajectories, over and above the variation explained by included covariates. Differences in weight and length trajectories were observed between the clusters; the more breastfeeding that was included in a cluster, the closer the growth pattern resembled that of the breastfed reference group. No distinct differences in BMI trajectories were apparent within the first year of life. Mean weight-for-age *z*-scores per cluster all fell within ±0.5 SD of the WHO standard, indicative for adequate growth.

**Conclusions:**

The results of this study suggest that our previously defined milk feeding clusters can discriminate growth trajectories between the different feeding groups in the studied population. This confirms that our proposed approach has the potential to bring more precision to future studies examining associations between milk feeding patterns and growth (and potentially other health) outcomes in the first year of life across different populations. Conclusions on a causal effect of feeding characteristics should be made with caution because of the observational and exploratory nature of the analysis.

This trial was registered at clinicaltrials.gov as NCT01609634.

## Introduction

Because of the undeniable benefits of breastfeeding (BF) for both mother and child, the WHO recommends exclusive BF (EBF) for the first 6 mo, with introduction of complementary foods and continued BF thereafter [1]. However, if parents cannot or choose not to provide EBF for any reason, infants may be fed infant formula (IF) and/or follow-on formula in addition to breastmilk. This mode of infant feeding, where both BF and formula feeding (FF) are provided to an infant during the same period and is often referred to as combination feeding, will be referred to as mixed milk feeding (MMF) in this article.

Global MMF prevalence rates during the first 2 years of life have been estimated at 23%–32% [2]. Our previous research, using data from 2 randomized controlled trials (RCTs) in Europe and Asia, found MMF prevalence during the first year in these studies to be close to 50%, suggesting it to be a common feeding practice [3]. Despite this substantial proportion of infants receiving MMF, there is limited research on the association between MMF and growth and other (health related) outcomes, in particular with MMF according to different proportions or durations [3].

MMF encompasses a highly heterogenous group of feeding behaviors that vary widely in terms of the proportions of breastmilk and IF provided, the timing of IF introduction, the total duration of BF, MMF, and FF, the composition of the formula given, and how these factors evolve. This variability makes it challenging to standardize MMF over time and to measure its associations with health-related outcomes, such as growth. This may explain the limited number of studies specifically investigating the associations between MMF and growth and other (health) outcomes. Additionally, differing and inconsistent classifications and definitions of terms such as “MMF,” “combination feeding,” and “mixed feeding” [3,4] make comparisons across studies often unreliable.

In a previous publication, we described a new approach for studying MMF patterns across the first year of life. By using a statistical multivariate clustering algorithm applied to individual participant data from a clinical study (*n* = 855, an RCT conducted predominantly in European infants aged 0–12 mo and at risk of allergy; clinicaltrials.gov registry identifier: NCT03067714), we structured MMF data and identified groups of subjects with similar observed feeding patterns over time. Four distinct MMF clusters were identified: early exclusive FF (32%), later exclusive FF (25%), long-term MMF (21%), and mostly BF (22%) [3]. We then validated the robustness of the outcomes of the applied statistical method (reported in the same publication) by reproducing the results in a second clinical study, the Venus study, conducted in a different world region (*n* = 539, an RCT carried out in a population of healthy Singaporean infants) [5].

The proposed methodology can significantly enhance infant feeding research by providing a starting point for studying the impact of various milk feeding patterns on health and other outcomes. It can help evaluate whether MMF, particularly in relation to specific timings, or IF volumes, offers health benefits over exclusive FF. The associations between MMF, especially according to different patterns throughout the first year, and health outcomes such as brain and immune development are still poorly understood. However, these associations could be researched more precisely and consistently by applying such feeding clusters.

Building on our previous work [3], where we defined and tested the reproducibility of our newly described MMF clustering methodology, the current study assessed whether these clusters can successfully discriminate different growth patterns—by feeding cluster—across the first year of life. To achieve this, we used data from the above-mentioned Venus clinical study, where equivalence in daily weight gain at 4 mo was the primary outcome measure, and examined associations between the different MMF clusters and growth outcomes during the first year of life.

## Methods

### Clinical study description

The Venus study aimed to evaluate if a Concept IF (large lipid droplets with a coating of milk fat globule membrane component and containing a specific mixture of prebiotics) resulted in equivalent growth adequacy and safety outcomes compared with standard IF during the first 4 mo of life, in healthy, term Singaporean infants. In total, 520 subjects (of Chinese, Malay, or Indian ethnicity) were either randomly assigned or included in the BF reference group. The study was registered in the clinicaltrials.gov registry (identifier: NCT01609634) and allowed infants younger than 28 d of age to participate, regardless of their feeding method. Infants were randomly assigned to 1 of 3 groups only after parents decided to introduce FF: the test IF or 1 of 2 control products. All formulas were isocaloric (66 kcal/100 mL) and contained similar amounts of protein (1.3 g/100 mL) and lipids. Infants exclusively BF at enrolment, whose mothers intended to continue EBF until at least 3 mo of age and did not start FF before 4 mo of age were included in the study’s BF reference group.

After informed consent was obtained, infants’ demographic information, anthropometric data at birth, and medical history were collected from medical records by study staff during the enrolment visit. At the next study visit, additional information on parental family and household characteristics, including anthropometric and substance use data, were gathered from parents during an interview using a standardized form. Anthropometric measurements were conducted during the infant’s first year at the ages of 4, 8, 13, 17, 26, 39, and 52 wk by trained study personnel following standardized procedures. Feeding data were collected through a 7-d diary completed by the parents before each visit, which included the daily volume of study formula intake and the number of daily feedings of both study product and breastmilk. Questionnaires were administered during in-person visits, capturing details such as start and stop ages of BF, study formula use, other IF consumption, and the introduction of complementary feeding. Further details of the study, including study products tested, and safety and tolerance outcomes can be found in the studies by Shek et al. [5] and Teoh et al. [6].

### MMF clustering approach previously described

A detailed description of the methods applied to define 6 feeding clusters is reported by Papadopoulos et al. [3]. Briefly, all subjects who received MMF for ≥21 d between birth and 1 y of age were included in the MMF subset. A day of MMF was defined as the consumption of both breastmilk and IF on the same day, regardless of the proportion of each, the type of IF, or the presence of complementary feeding. Infants with either minimal breastmilk or minimal IF intake recorded during the first year (on ≤5% of total recorded days) were categorized as Ref-BF (breastfeeding reference) or formula feeding reference (Ref-FF), respectively.

A multivariate k-means clustering algorithm [7] was applied to assign subjects in the MMF subset into 4 distinct MMF groups, or clusters, based on the timing of IF introduction and the duration of BF and the volume of IF received over time, or into reference feeding clusters (Ref-BF and Ref-FF). The MMF clusters were named as follows: early exclusive FF, later exclusive FF, long-term MMF, and mostly breastfeeding. The approach we followed for the current research was the same as the one described earlier except that it facilitated inclusion of a slightly larger number of subjects by not placing restrictions on the maximum follow-up time.

### Baseline demographics and complementary feeding

Baseline and demographic variables collected as part of the clinical study, including birth weight, ethnicity, and sex, were summarized and compared descriptively by cluster because of their potential relevance for growth. Additionally, because the timing of complementary feeding could influence length and weight development, we compared the mean timing of the introduction of complementary foods by cluster.

### Testing the associations between the feeding clusters and growth

To analyze the relationship between the feeding clusters (including reference groups) and various growth outcomes during the first year of life, a linear mixed-effects model (parametric growth curve model) was used. Six growth outcomes were analyzed, with the raw outcome value used as the dependent variable in the model: BMI (in kg/m^2^; and BMI-for-age *z*-score), length (centimeters; and length-for-age *z*-score), and weight (kilograms; and weight-for-age *z*-score). Growth measure *z*-scores were determined according to the WHO Child Growth Standards, specific for age and sex [8,9]. The linear mixed-effects model included random intercepts and linear and quadratic slopes of age for each subject to capture individual variability. For the fixed-effects part, natural cubic splines were used to flexibly model age effects. Three internal knots were used, placed at 1, 6, and 9 mo. Interaction effects between age splines and the feeding clusters were used to capture cluster-specific growth trajectories. Feeding cluster was modeled as an ordered factor, reflecting the progression from predominantly BF to predominantly FF. The model included main effects of ethnicity, birth length, birth weight, and sex. Additionally, to correct for their effects over time, interactions of these confounding factors (ethnicity, birth length, birth weight, and sex) with both linear and quadratic age were included.

The specification of the mixed-effects model is as follows:

$$y_{i}\left(t\right)=\beta_{0}+\beta_{1}B_{n}\left(t,\lambda_{1}\right)+\beta_{2}B_{n}\left(t,\lambda_{2}\right)+\beta_{3}B_{n}\left(t,\lambda_{3}\right)+\beta_{4}B_{n}\left(t,\lambda_{4}\right)+\beta_{5}\left[B_{n}\left(t,\lambda_{1}\right)\;\cdot {cluster}_{i}\right]+\beta_{6}\left[B_{n}\left(t,\lambda_{2}\right)\cdot {cluster}_{i}\right]+\beta_{7}\left[B_{n}\left(t,\lambda_{3}\right)\cdot {cluster}_{i}\right]+\beta_{8}\left[B_{n}\left(t,\lambda_{4}\right)\cdot {cluster}_{i}\right]+\beta_{9}{\;ethnic}_{i}+\beta_{10}\;{brthwght}_{i}+{\beta_{11}\;{brthlen}_{i}+\beta }_{12}\;{sex}_{i}+\beta_{13}{\;ethnic}_{i}\cdot t+\beta_{14}\;{brhwght}_{i}\cdot t+{\beta_{15}\;{brthlen}_{i}\cdot t+\beta }_{16}\;{sex}_{i}\cdot t+\beta_{17}{\;ethnic}_{i}\cdot t^{2}+\beta_{18}\;{brhwght}_{i}\cdot t^{2}+{\beta_{19}\;{brthlen}_{i}\cdot t^{2}+\beta }_{20}\;{sex}_{i}*t^{2}+b_{i0}+b_{i1}t+b_{i2}t^{2}$$

where *t* is time since birth (ie, age); $y_{i}$ is the growth outcome for subject *i*; $\left\{B_{n}\left(age,\lambda_{k}\right):k=1,2,3,4\right\}$ denotes the B-spline basis matrix for a natural cubic spline of age with 3 internal knots; and $b_{i0}$, $b_{i1}\text{,}$ and $b_{i2}$ are the random intercept, random slope, and random quadratic slope, respectively.

The splines approach has the advantage of allowing for nonlinear evolutions over time while also accommodating simple linear relationships. In our primary model, we chose not to include the main effect of feeding cluster, as baseline differences could be misinterpreted as if these clusters reflect fixed, pre-existing conditions from baseline onward. Feeding clusters, instead, are defined based on feeding patterns over the entire first year, not by feeding status at baseline. To assess the robustness of our findings, we conducted a sensitivity analysis including the main effect of feeding cluster. The results of this analysis are presented in Supplemental Appendix 1.

Linear mixed-effects models were fitted using the statistical software package R using the lme4 package. We first fitted these mixed-effects models to the actual data. Then, based on these models, we predicted a value for each outcome at each time point for all individuals in the data, as if every individual existed in every feeding cluster. This allowed us to estimate the predicted mean outcome for each cluster over time by averaging across individuals. This approach, known as model-based standardization [10], enabled us to compare growth trajectories across feeding clusters while holding confounding factors constant. We used a model-based semiparametric bootstrap approach to estimate 95% CIs. For each bootstrap iteration, the mixed-effects model was refitted on a resampled version of the original data set. The fitted model was then used to generate predictions for the standardized data set in which all individuals were assigned to each feeding cluster. This approach accounts for uncertainty in both fixed and random effects without assuming a specific distribution for the outcome.

The predicted mean difference (compared with BF-Ref subjects) was calculated by averaging the differences between the fitted values in a given cluster and the fitted values in the Ref-BF cluster. We performed a likelihood ratio test by comparing the log-likelihoods of the mixed-effects model with and without the terms involving the feeding clusters. Both models were refitted using maximum likelihood estimation to ensure valid comparison. This allowed us to assess whether the inclusion of feeding cluster terms significantly improved the model fit. We repeated this analysis for the sensitivity model that included the main effect of feeding, for which the results are presented in Supplemental Appendix 2.

## Results

### Cluster groups and demographic, baseline, and subjects variables

The group sizes used to investigate the associations between feeding clusters and growth are shown in Table 1. Overall, 467 infants were included in cluster analysis (MMF and reference feeding clusters). The largest MMF cluster was classified as early exclusive FF (29.8%), followed by the long-term MMF cluster (15.2%). The treatment groups assigned to FF subjects were evenly distributed between the clusters.

**TABLE 1:** Number and percentage of infants in each of the MMF and reference clusters

Milk feeding cluster	Group size, *n* (%)
Total	467 (100)
BF-Ref	36 (7.7)
Mostly BF	66 (14.1)
Long-term MMF	71 (15.2)
Later exclusive FF	65 (13.9)
Early exclusive FF	139 (29.8)
FF-Ref	90 (19.3)

Abbreviations: BF, breastfed; FF, formula feeding; MMF, mixed milk feeding; Ref, reference.

Demographic and baseline variables and the timing of introduction of complementary feeding are shown in Table 2, distributed by cluster. The proportion of mothers with at least a university education was higher in the Ref-BF (83.3%) and mostly BF (65.2%) clusters than that in the other clusters (all <40%, with the Ref-FF cluster being the lowest at 10%). Correspondingly, in the Ref-BF and mostly BF clusters, infants came from households falling predominantly in the highest income category (72.2% and 68.2%, respectively) compared with the other clusters (ranging between 46.2% in the later exclusive FF cluster and 10% in the Ref-FF cluster). There was also a higher proportion of Chinese infants included in the Ref-BF (94.4%) and mostly BF (86.4%) clusters, than that in the Ref-FF (46.7%) and early FF (51.8%) clusters. Infants in the Ref-BF cluster were born on an average of 88 g heavier than infants in the Ref-FF cluster (mean birth weight: 3.201 kg; SD: 0.350 kg; compared with mean birth weight: 3.113 kg; SD: 0.353 kg). In the mostly BF cluster, girls were more prevalent (56.1%) than boys (43.9%). Conversely, the later exclusive FF and Ref-FF clusters had more boys (61.5% and 60%, respectively) than girls (38.5% and 40%, respectively). In the remaining clusters, the sex distribution was relatively balanced. The timing of the introduction of complementary feeding was evenly distributed between the clusters.

**TABLE 2:** Demographic, baseline, and subject variables, by cluster

	Ref-BF (*n* = 36)	Mostly BF (*n* = 66)	Long-term MMF (*n* = 71)	Later exclusive FF (*n* = 65)	Early exclusive FF (*n* = 139)	Ref-FF (*n* = 90)	Total (*N* = 467)
Parental demographic and baseline variables							
Highest level of education to date, biological mother							
No university	6 (16.7)	23 (34.8)	47 (66.2)	38 (58.5)	100 (71.9)	81 (90.0)	295 (63.2)
University or other higher	30 (83.3)	43 (65.2)	24 (33.8)	27 (41.5)	39 (28.1)	9 (10.0)	172 (36.8)
Net income of the household per month (SGP dollars)							
<2500	2 (5.6)	3 (4.5)	16 (22.5)	6 (9.2)	32 (23.0)	50 (55.6)	109 (23.3)
2500–5500	8 (22.2)	18 (27.3)	24 (33.8)	29 (44.6)	51 (36.7)	26 (28.9)	156 (33.4)
>5500	26 (72.2)	45 (68.2)	31 (43.7)	30 (46.2)	56 (40.3)	14 (15.6)	202 (43.3)
Age (y), biological mother							
Mean (SD)	30.1 (3.9)	31.4 (3.9)	32.1 (4.8)	30.3 (4.4)	30.5 (5.0)	29.2 (6.1)	30.6 (4.9)
Range	19–38	21–44	20–44	17–39	20–42	18–43	17–44
Prepregnancy BMI (kg/m^2^), biological mother							
Mean (SD)	20.9 (2.5)	21.8 (3.2)	23.2 (4.6)	22.2 (5.0)	23.1 (5.0)	24.1 (6.3)	22.8 (4.9)
Range	16.9–28.2	16.5–31.6	15.1–35.6	15.8–44.1	15.2–37.7	15.4–47.9	15.1–47.9
Height (cm), biological father/legal guardian							
Mean (SD)	171.7 (6.7)	172.7 (6.4)	172.5 (5.6)	173.1 (5.1)	171.8 (6.2)	170.9 (6.5)	172.0 (6.1)
Range	153–183	157–184	160–181	162–190	157 – 19	150–190	150–193
Height (cm), biological mother							
Mean (SD)	160.0 (5.6)	159.6 (5.3)	159.4 (5.5)	160.0 (5.6)	159.2 (5.6)	158.1 (6.5)	159.2 (5.7)
Range	150–175	147–170	147–173	148–170	143–174	140–174	140–175
BMI (kg/m^2^), biological father/legal guardian							
Mean (SD)	24.8 (4.0)	24.7 (3.4)	25.1 (3.9)	25.0 (4.6)	25.3 (4.6)	25.6 (5.2)	25.1 (4.4)
Range	16.6–38.1	18.0–33.1	19.1–41.0	17.9–36.3	17.4–45.0	18.6–49.4	16.6–49.4
Maternal diabetes							
No	33 (91.7)	59 (89.4)	64 (90.1)	61 (93.8)	128 (92.1)	86 (95.6)	431 (92.3)
Yes	3 (8.3)	7 (10.6)	7 (9.9)	4 (6.2)	11 (7.9)	4 (4.4)	36 (7.7)
Antibiotics used during delivery?							
No	27 (75.0)	53 (80.3)	52 (73.2)	45 (69.2)	105 (75.5)	75 (83.3)	357 (76.4)
Yes	9 (25.0)	13 (19.7)	19 (26.8)	20 (30.8)	34 (24.5)	15 (16.7)	110 (23.6)
Infant baseline and demographic variables							
Gestational age (wk)							
Mean (SD)	39.2 (1.2)	39.0 (1.0)	39.1 (1.0)	39.0 (0.9)	38.9 (1.0)	38.8 (1.0)	39.0 (1.0)
Range	37.2–41.9	37.0–40.9	37.0–40.7	37.1–40.6	37.0–41.0	37.0–41.3	37.0–41.9
Sex							
Female	19 (52.8)	37 (56.1)	38 (53.5)	25 (38.5)	72 (51.8)	36 (40.0)	227 (48.6)
Male	17 (47.2)	29 (43.9)	33 (46.5)	40 (61.5)	67 (48.2)	54 (60.0)	240 (51.4)
Mode of delivery							
Cesarean	6 (16.7)	14 (21.2)	17 (23.9)	17 (26.2)	38 (27.3)	22 (24.4)	114 (24.4)
Vaginal (including instrumental)	30 (83.3)	52 (78.8)	54 (76.1)	48 (73.8)	101 (72.7)	68 (75.6)	353 (75.6)
Birth weight (kg)							
Mean (SD)	3.201 (0.350)	3.183 (0.310)	3.198 (0.360)	3.211 (0.296)	3.125 (0.383)	3.113 (0.353)	3.160 (0.351)
Range	2.545–3.846	2.410–3.900	2.440–4.055	2.470–3.762	2.120–4.122	2.435–4.162	2.120–4.162
Birth length (cm)							
Mean (SD)	49.806 (2.202)	49.515 (1.939)	49.268 (1.804)	49.523 (1.855)	48.986 (1.978)	48.500 (1.730)	49.148 (1.935)
Range	46.000–58.000	46.000–54.000	45.000–53.000	45.000–53.000	43.000–55.000	44.000–55.000	43.000–58.000
Birth weight ≥85th WHO percentile							
No	36 (100)	64 (97)	68 (96)	65 (100)	131 (94)	88 (98)	452 (97)
Yes	0 (0)	2 (3)	3 (4)	0 (0)	8 (6)	2 (2)	15 (3)
Ethnicity							
Chinese	34 (94.4)	57 (86.4)	50 (70.4)	48 (73.8)	72 (51.8)	42 (46.7)	303 (64.9)
Non-Chinese	2 (5.6)	9 (13.6)	21 (29.6)	17 (26.2)	67 (48.2)	48 (53.3)	164 (35.1)
Timing of introduction of complementary feeding (mo)							
Median (95% CI)	5.7 (5.5, 5.9)	5.7 (5.5, 5.9)	5.7 (5.3, 5.9)	5.5 (5.2, 5.9)	5.5 (5.3, 5.8)	5.5 (5.2, 5.9)	5.6 (5.5, 5.7)

Values are *n* (%) unless specified.

Abbreviations: BF, breastfed; FF, formula feeding; MMF, mixed milk feeding; Ref, reference.

### Associations between feeding clusters and growth outcomes

#### Weight and weight-for-age z-scores by feeding cluster

**FIGURE 1:**
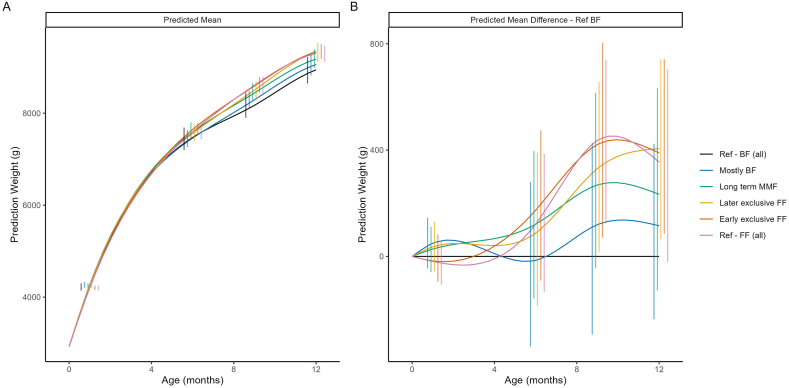
Model-based predicted mean weight (A) and predicted mean difference in weight compared with the breastfed reference (Ref-BF) cluster (B). The model included main effects of ethnicity, birth length, birth weight and sex, as well as their interactions with both linear and quadratic age. Error bars represent 95% CIs at selected time points. BF, breastfed; FF, formula feeding; MMF, mixed milk feeding; Ref, reference.

**FIGURE 2:**
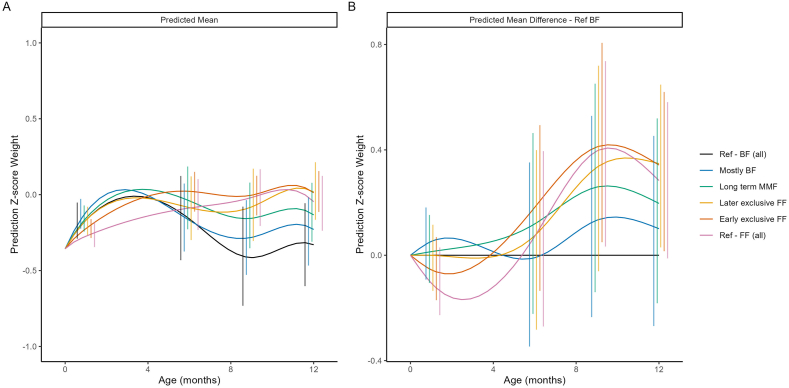
Predicted mean weight-for-age *z*-score (A) and predicted mean difference in weight-for-age *z*-score compared with the breastfed reference (Ref-BF) clusters (B). The model included main effects of ethnicity, birth length, birth weight and sex, as well as their interactions with both linear and quadratic age. Error bars represent 95% CIs at selected time points. BF, breastfed; FF, formula feeding; MMF, mixed milk feeding; Ref, reference.

Figure 1 shows the predicted mean weight and the difference in predicted mean weight trajectories compared with Ref-BF, by cluster. The highest overall weight gain compared with the Ref-BF cluster was observed in the Ref-FF and early exclusive FF clusters, whose weight trajectories were similar, followed by the later exclusive FF cluster. Notably, the order of the clusters suggests a dose effect: the more BF in a cluster, the closer the growth pattern resembled that of the Ref-BF cluster. The average growth in weight and mean weight-for-age *z*-score for the Ref-BF cluster was similar to the other clusters until ∼6 mo of age, after which it became relatively lower. Mean weight-for-age *z*-scores for all clusters fell within ±0.5 SD of the WHO reference standards (Figure 2).

#### Length and length-for-age z-score by feeding cluster

**FIGURE 3:**
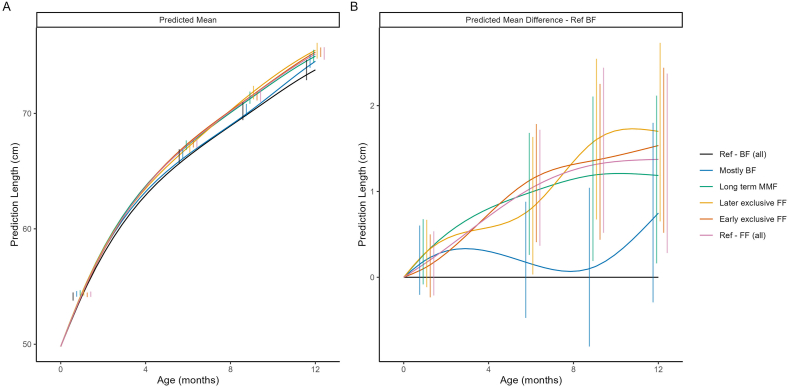
Model-predicted mean length (A) and predicted mean difference in length compared with the breastfed reference (Ref-BF) cluster (B). The model included main effects of ethnicity, birth length, birth weight and sex, as well as their interactions with both linear and quadratic age. Error bars represent 95% CIs at selected time points. BF, breastfed; FF, formula feeding; MMF, mixed milk feeding; Ref, reference.

**FIGURE 4:**
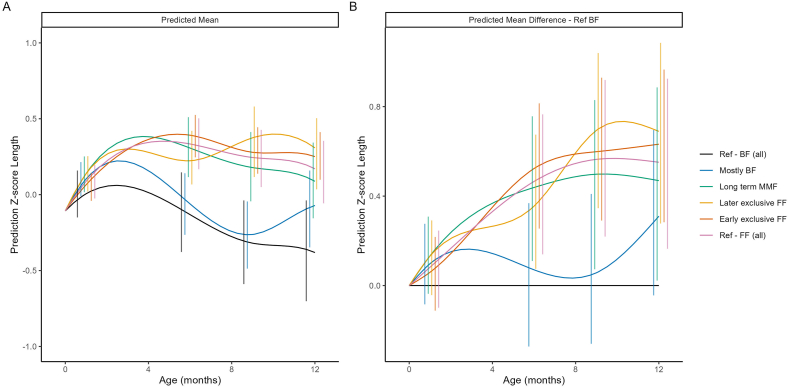
Predicted mean *z*-length (A) and predicted mean difference in *z*-length compared with the breastfed reference (Ref-BF) group (B). The model included main effects of ethnicity, birth length, birth weight and sex, as well as their interactions with both linear and quadratic age. Error bars represent 95% CIs at selected time points. BF, breastfed; FF, formula feeding; MMF, mixed milk feeding; Ref, reference.

Figure 3 shows the predicted mean length and the difference in predicted mean length trajectories compared with Ref-BF, by cluster. Mean length-for-age *z*-scores for the MMF clusters were within ±0.5 SD of the WHO reference standards (Figure 4). Consistent with our observations on weight, the Ref-FF, early exclusive FF and later exclusive FF clusters had the highest length, followed by the Long-term MMF, mostly BF, and Ref-BF clusters (FIGURE 3, FIGURE 4). Except for the mostly BF cluster, which showed length values comparable with those of the Ref-BF cluster, all other feeding clusters had substantially higher length values than those of the Ref-BF cluster. The average length patterns for all clusters were within the normal ranges.

#### BMI and WHO BMI-for-age z-scores by feeding cluster

**FIGURE 5:**
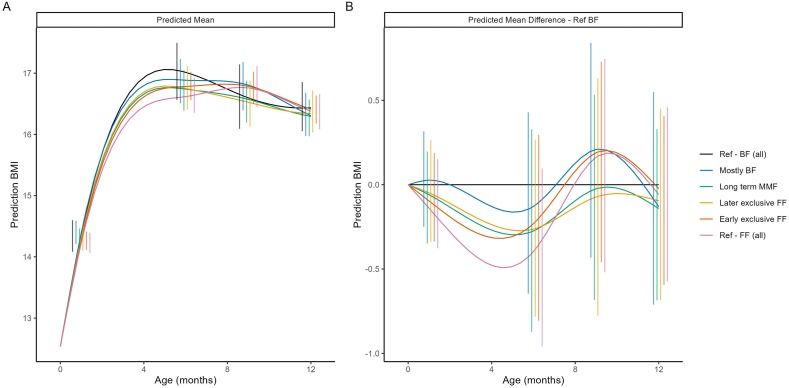
Model-predicted mean BMI (A) and predicted mean difference in BMI compared with the breastfed reference (Ref-BF) cluster (B). The model included main effects of ethnicity, birth length, birth weight and sex, as well as their interactions with both linear and quadratic age. Error bars represent 95% CIs at selected time points. BF, breastfed; FF, formula feeding; MMF, mixed milk feeding; Ref, reference.

**FIGURE 6:**
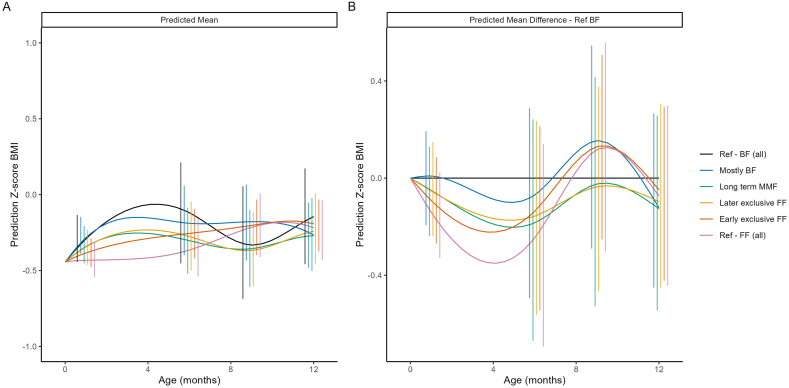
Predicted mean WHO BMI-for-age *z*-score (A) and predicted mean difference in WHO BMI-for-age *z*-score compared with the breastfed reference (Ref-BF) cluster (B). The model included main effects of ethnicity, birth length, birth weight and sex, as well as their interactions with both linear and quadratic age. Error bars represent 95% CIs at selected time points. BF, breastfed; FF, formula feeding; MMF, mixed milk feeding; Ref, reference.

No sizable differences were observed between the feeding clusters for BMI, particularly by the end of the first year (FIGURE 5, FIGURE 6), as emphasized by the largely overlapping 95% CIs at each time point. This result is not surprising given the similar patterns in both weight and length across the clusters. Where average weight was higher, average length was correspondingly higher, resulting in proportional growth trajectories. Consequently, the differences in BMI patterns were minimal.

The results of the sensitivity analysis including the main effect of feeding are shown in Supplemental Figures 1–6. As can be seen, incorporating cluster main effects did not change our findings. After ∼3 mo of age, the predicted growth patterns for each cluster remained virtually identical to those in the model without the main effect.

### Variation in growth trajectories explained by the clustering approach

Table 3 shows the *P* values when comparing the log-likelihoods of the mixed-effects model with and without the terms involving the feeding clusters. Comparing the log-likelihoods of the mixed-effects model with and without the terms involving the feeding clusters, it can be seen that the model fit for weight, length, weight-for-age *z*-score, and length-for-age *z*-score improved significantly (*P* < 0.001). This improvement was not observed for BMI and BMI-for-age *z*-score. These results indicate that the cluster division has added value by explaining a significant amount of variation in growth trajectories, beyond the variation already accounted for by the covariates (i.e., ethnicity, birth length, birth weight, and sex). The results of the analysis including the main effect of feeding are shown in Supplemental Table 1 in Supplemental Appendix 2.

**TABLE 3:** Test statistics and *P* values when comparing a full mixed-effects model including feeding cluster interactions against a null model excluding all feeding cluster terms

Outcome	Test statistic (χ^2^)	df	*P*	Outcome	Test statistic (χ^2^)	df	*P*
Weight	84.078	20	<0.001	*z*-Weight	82.09	20	<0.001
Length	54.711	20	<0.001	*z*-Length	52.968	20	<0.001
BMI	25.647	20	0.177	*z*-BMI	27.711	20	0.116

## Discussion

This study highlights the potential impact of specific milk feeding practices on infant growth throughout the first year. We compared growth trajectories in infants grouped by different milk feeding patterns, as described in the study by Papadopoulos et al. [3]. This approach allowed us to account for the high variability inherent in MMF behaviors and data. Unlike previous studies that categorized infants based on a single point in time or used less precise groupings, our study used more granular methods to investigate the relationships between growth trajectories and feeding characteristics in the first year of life.

The milk feeding cluster division explained a statistically significant amount of variation in growth trajectories, beyond what was already accounted for by the included covariates. The method successfully discriminated between different growth trajectories over time. In addition, when MMF patterns were organized by BF duration, our study indicated a dose effect. Infants grouped into clusters with longer (exclusive) BF had average weight and length patterns more similar to the Ref-BF group, whereas those grouped into clusters with longer FF durations and higher FF intakes had weight and length patterns closer to the Ref-FF group from 6 to 12 mo of age. This is a plausible finding, bringing further reliability to the results. The body weight and length development of the long-term MMF cluster infants fell between those who were exclusively FF and EBF.

Overall, the average weight, length, and BMI for each MMF cluster were comparable with WHO standards for healthy infant growth. Consistent with other research, FF infants were heavier and/or longer than BF infants between 6 and 12 mo of age [11,12]. The lower average weight and length seen in the Ref-BF and mostly BF clusters than the WHO reference standards may be because of the genetic context of the Singaporean population studied. An article reviewing the growth of Asian infants reported that postnatal growth trajectories are different between countries within and outside Asia [13]. A similar result has been seen in Japanese infants. In a study of 647 children, those who were BF were significantly lighter and shorter throughout most of the first 24 mo than the WHO (as well as national) children standards [14]. The authors postulate epigenetic factors as a possible reason, noting also that no samples from populations in East Asia were included in the WHO multicenter growth reference study [9,15].

The absence of BMI differences in our model by the end of 12 mo can be explained by the fact that both average weight and length were proportionally higher or lower across the different clusters. In addition, it could be attributable to various factors, such as differences in feeding and/or weaning practices, genetic predispositions, or environmental influences not accounted for that may have an impact on BMI. This highlights the importance of considering multiple growth parameters and influencing factors, as well as conducting longer-term follow-up studies to fully understand the implications of early infant feeding practices on growth and obesity risk. Some published research investigates the relationship between growth and exclusive compared with nonexclusive BF, but it often remains unclear what was fed in addition to breastmilk in the nonexclusive BF group (FF, animal milks, or other drinks/foods besides FF), leaving the influence of MMF with IF specifically unclear [16]. In some studies, the growth of infants fed both breastmilk and IF has been investigated. However, these studies often lack precise grouping of infants into feeding classes or do not define their feeding groups with adequate accuracy. For instance, feeding data may be collected cross-sectionally or via 24-h recall measures, often not capturing data on the durations of BF/MMF or volumes of IF given [17]. MMF infants may often be grouped into 1 category regardless of how long they were MMF or the ratios of FF to BF given [[18], [19], [20], [21], [22]]. Finally, some studies combine MMF and EBF infants into 1 BF group, whereas in other studies, MMF infants are considered part of the FF group or are even excluded from the analysis altogether [[23], [24], [25]]. This inconsistency in the classification of MMF may explain the mixed results in literature when comparing the growth of MMF infants with EBF or FF infants in the first year.

A systematic review of 20 studies found inconsistent outcomes regarding the impact of BF duration on growth, noting that “shorter partial BF duration was associated with higher weight gain during infancy, with limited or inconclusive data on other growth parameters” [26]. Several studies have suggested that longer BF durations are linked to more favorable BMI outcomes than shorter durations. For instance, a study of >500 Australian children found that BF, whether exclusive or not, for ≥6 mo compared with <6 mo, was associated with more favorable BMI WHO-based *z*-score trajectories [27]. Another recent systematic review [28] reported that most cohort studies consistently found associations between longer BF duration (both any and exclusive) and lower BMI trajectories up to the age of 18 y. Conversely, an analysis of data from >7000 Chinese infants aged 3 mo to 16 y revealed similar BMI-for-age *z*-score trajectories for those exclusively BF, MMF, or exclusively FF at 3 mo [22]. The differences in how infant milk feeding modes and MMF are classified may have contributed to these conflicting findings [29]. Using a more robust methodology, as used in the current study, to categorize infants into different feeding groups where the data permit, will facilitate easier and more reliable comparisons across studies.

Our findings suggest that MMF throughout the first year, rather than switching entirely to FF, may result in bringing growth patterns closer in line with EBF infants. A study in 70,000 Japanese children similarly concluded, “breastfeeding, even if partial or for short duration, has a latent protective effect against overweight and obesity in late childhood” [30]. This suggests that, when EBF is not possible, even brief durations of BF, or BF while supplementing with FF, can positively impact infant growth. It has been reported that mothers who practice MMF may experience guilt more than mothers who practice EBF [31]. Understanding that any amount of breastmilk may still benefit infant development may provide encouragement and help alleviate these feelings of guilt. If our findings are confirmed, they could provide valuable reassurance to parents, encouraging them to continue BF alongside FF for as long as possible, instead of switching fully to FF.

To our knowledge, this study is the first to use a novel, sophisticated, data-driven method to group infants with similar milk feeding patterns over time and examine their association with growth. It is important to emphasize that our study compared nonrandomized feeding groups, which are inherently subject to both measured and unmeasured confounding factors. To mitigate this issue, we used a standardization approach that accounted for key measured confounders such as race, ethnicity, birth weight, and birth length. This approach enhances the validity of our findings by reducing the influence of confounders, allowing for a more accurate comparison of the feeding groups. However, it is important to acknowledge that both measured and unmeasured confounding may still be present. For instance, the clusters characterized by higher levels of BF were also associated with higher maternal education and household income than those with more FF. However, the model already included a complex structure, including several key confounders, random intercepts and linear and quadratic slopes, natural cubic splines for age, and multiple interaction terms to flexibly capture growth trajectories. Including additional variables such as socioeconomic status or maternal education would have further increased model complexity and risked overfitting, without necessarily improving causal interpretation. The included infants were part of an RCT in Singapore and, therefore, were offered the same access to health care regardless of their family’s socioeconomic status. In a more disparate population, the factors relating to household income or maternal education level may need more consideration for a similar analysis.

In addition to decisions around covariate inclusion, it is also important to acknowledge that using the standardization procedure means that any imbalances in or specific characteristics of the data are carried over. For example, if the population contains a larger proportion of a certain ethnic group, the predicted value for a given type of feeding refers to a hypothetical population with the same proportion of ethnic groups. This means that the results are specific to the current study population’s composition and may not be generalizable to populations with different demographic distributions.

Another methodological limitation is the potential for reverse causality, which arises from using feeding clusters based on data ≤1 y of age as an exposure. The various feeding choices made by parents may have been influenced by specific concerns about their infant’s growth, meaning the growth pattern could have influenced the feeding choice rather than the other way around [32]. Thus, the observed effects of feeding cluster represent the combined influence of feeding practices and other factors associated with these feeding practices. Similarly, complementary feeding patterns were not included in the analysis and may also be associated with milk feeding choice and/or growth. Therefore, it is important to note that, because of the observational nature of the study and the potential for residual confounding and reverse causality, the associations observed should not be interpreted as evidence of causal effects.

The implications of our findings and future research directions suggest that while the clustering approach is useful, it is computationally intense and requires statistical expertise. As a next step, it would be beneficial to develop a simplified approach of defining feeding clusters, based on the data-driven cluster grouping. Replicating our findings in other populations or in studies of longer duration and applying the methodology to discriminate other health outcomes based on different milk feeding patterns could be potentially relevant future research topics.

Overall, the results of the study reported in this article suggest that our previously defined milk feeding clusters can discriminate growth trajectories between different feeding groups in the studied population. This approach has the potential to bring more precision to future studies on infant feeding and the association between MMF patterns and health outcomes in the first year of life across different populations. The results could be used to build the evidence base to support health care practitioners in reassuring parents that prolonging BF brings benefits—with longer durations being associated with infant growth closer to that of EBF infants—even if the introduction of FF is inevitable. At the same time, conclusions on a causal effect of feeding characteristics should be made with caution because of the observational and exploratory nature of the analysis.

## Author contributions

The authors’ responsibilities were as follows – LFvdM: designed research; LPS, OHT: conducted research; FMvO: analyzed data and performed statistical analysis; LFvdM, KAM, FMvO, WWP: wrote the paper; LFvdM: had primary responsibility for final content; and all authors: have read and approved the final manuscript.

## Data availability

Data described in the manuscript and/or analytic code will be made available upon request, pending application and approval.

## Funding

Danone Research & Innovation (Utrecht, Netherlands) funded the study.

## Conflict of interest

LFvdM, KAM, and FMvO are employees of Danone Research & Innovation. All other authors declare no conflicts of interest.
