# Supplementary material for: Mixed Milk Feeding Patterns and Growth Outcomes During the First Year of Life in Asian Infants: Application of Predefined Feeding Clusters to Test Associations
Source: Curr Dev Nutr. 2025 Oct 3;9(11):107565. doi: 10.1016/j.cdnut.2025.107565 (PMC12581686; doi:10.1016/j.cdnut.2025.107565)
Supplement: Multimedia component 1 [file mmc1.docx]

**Supplementary Materials**

**APPENDIX 1**

**Associations between the feeding clusters and growth including cluster as main effect (Liandre Frances van der Merwe, et al.)**

Results

*Weight and weight-for-age z-scores by feeding cluster*

**
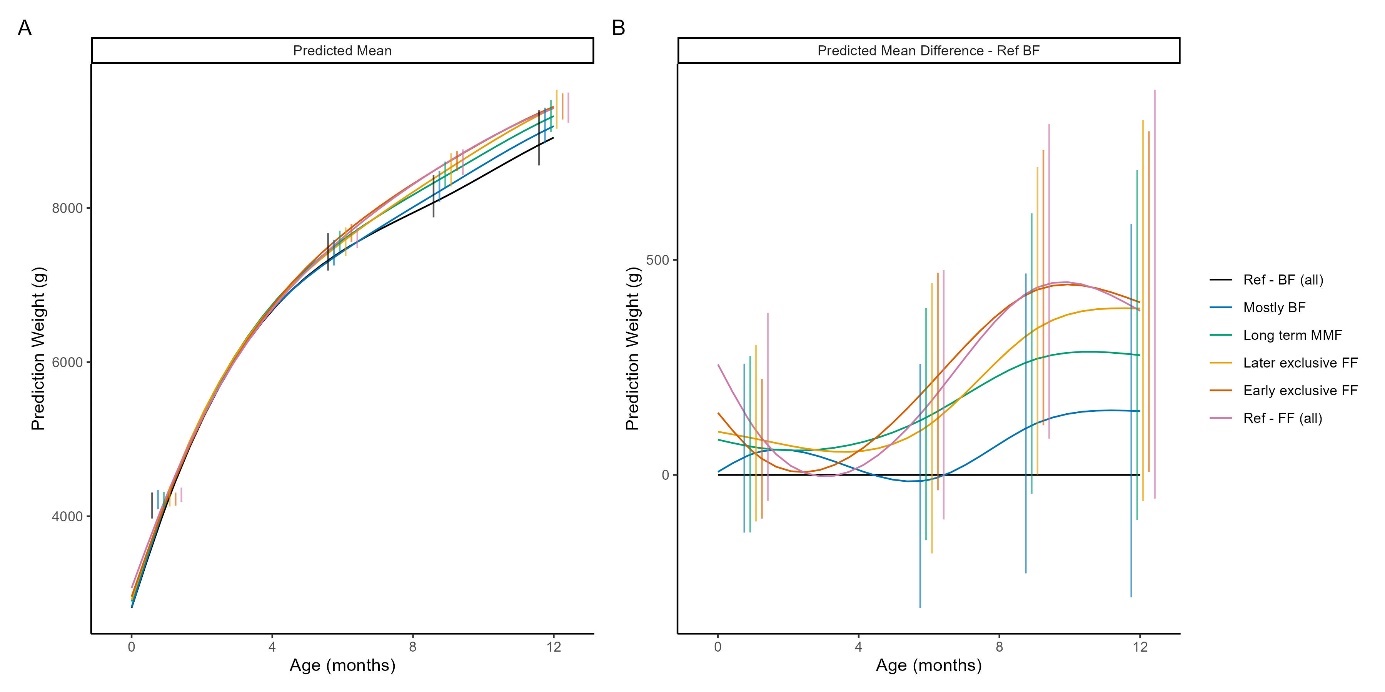
**

**Figure 1**: Model-based predicted mean weight (A) and predicted mean difference in weight compared to the breastfed reference (Ref-BF) cluster (B) for the model including the main effect of feeding cluster. The model also included main effects of ethnicity, birth length, birth weight and sex, as well as their interactions with both linear and quadratic age. Error bars represent 95% confidence intervals at selected timepoints.

^Abbreviations: BF: breastfed; FF: formula feeding; MMF: mixed milk feeding; Ref: reference^

**
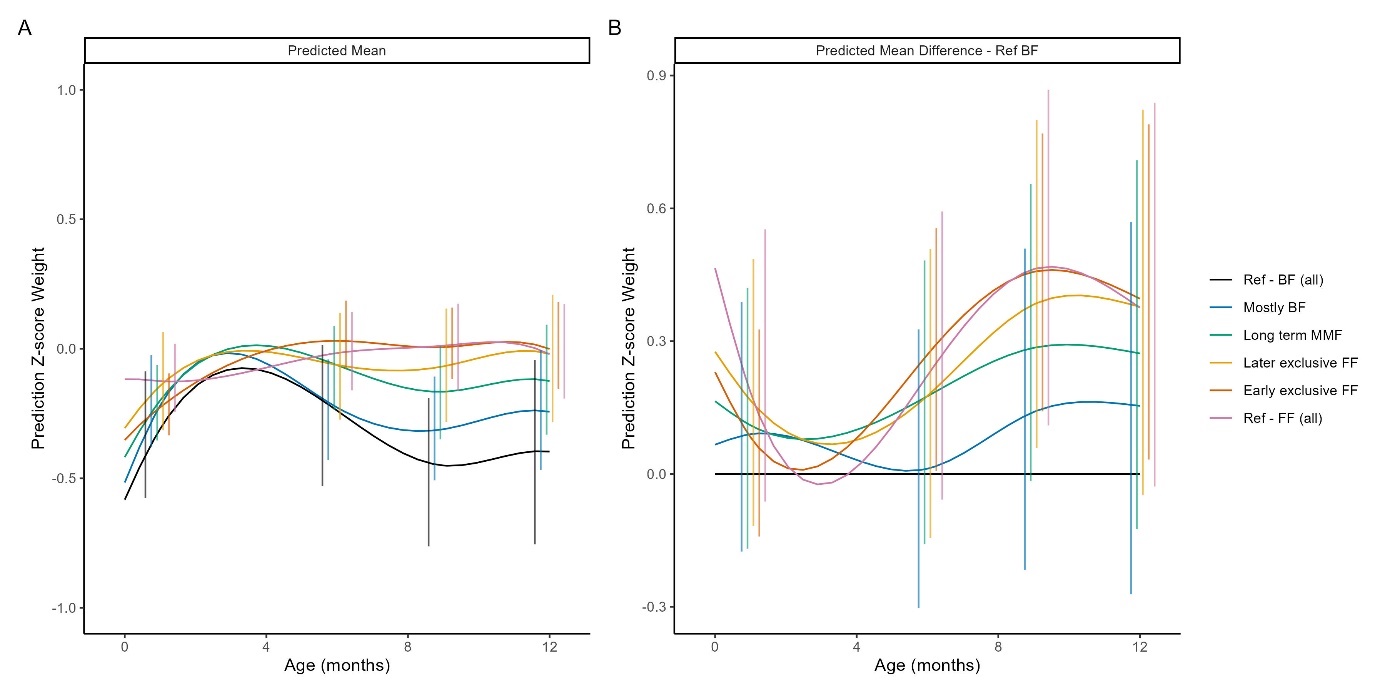
**

**Figure 2:** Predicted mean Weight-for-age z-score (A) and predicted mean difference in weight-for-age z-score compared to the Breastfed reference (Ref-BF) cluster (B) for the model including the main effect of feeding cluster. The model also included main effects of ethnicity, birth length, birth weight and sex, as well as their interactions with both linear and quadratic age. Error bars represent 95% confidence intervals at selected timepoints.

^Abbreviations: BF: breastfed; FF: formula feeding; MMF: mixed milk feeding; Ref: reference^

*Length and length-for-age z-score by feeding cluster*


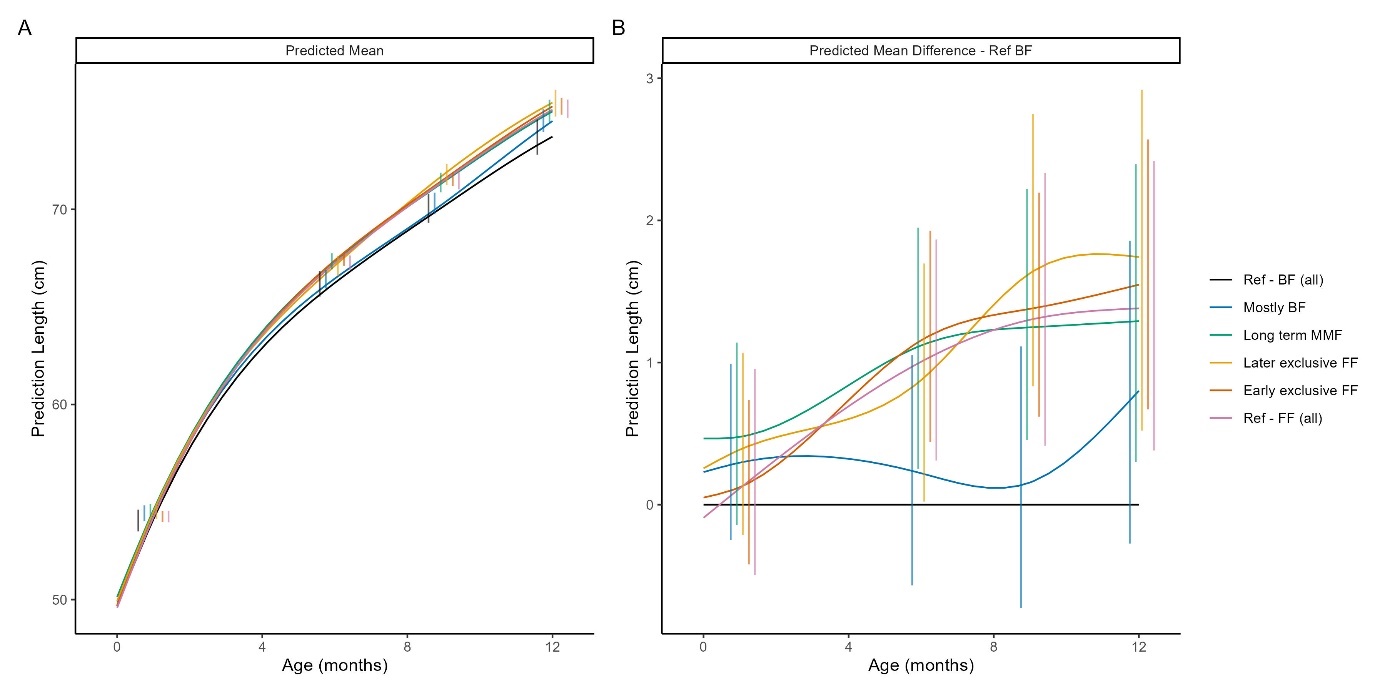


**Figure 3:** Model-predicted mean length (A) and predicted mean difference in length compared to the Breastfed reference (Ref-BF) cluster (B) for the model including the main effect of feeding cluster. The model also included main effects of ethnicity, birth length, birth weight and sex, as well as their interactions with both linear and quadratic age. Error bars represent 95% confidence intervals at selected timepoints.

^Abbreviations: BF: breastfed; FF: formula feeding; MMF: mixed milk feeding; Ref: reference^


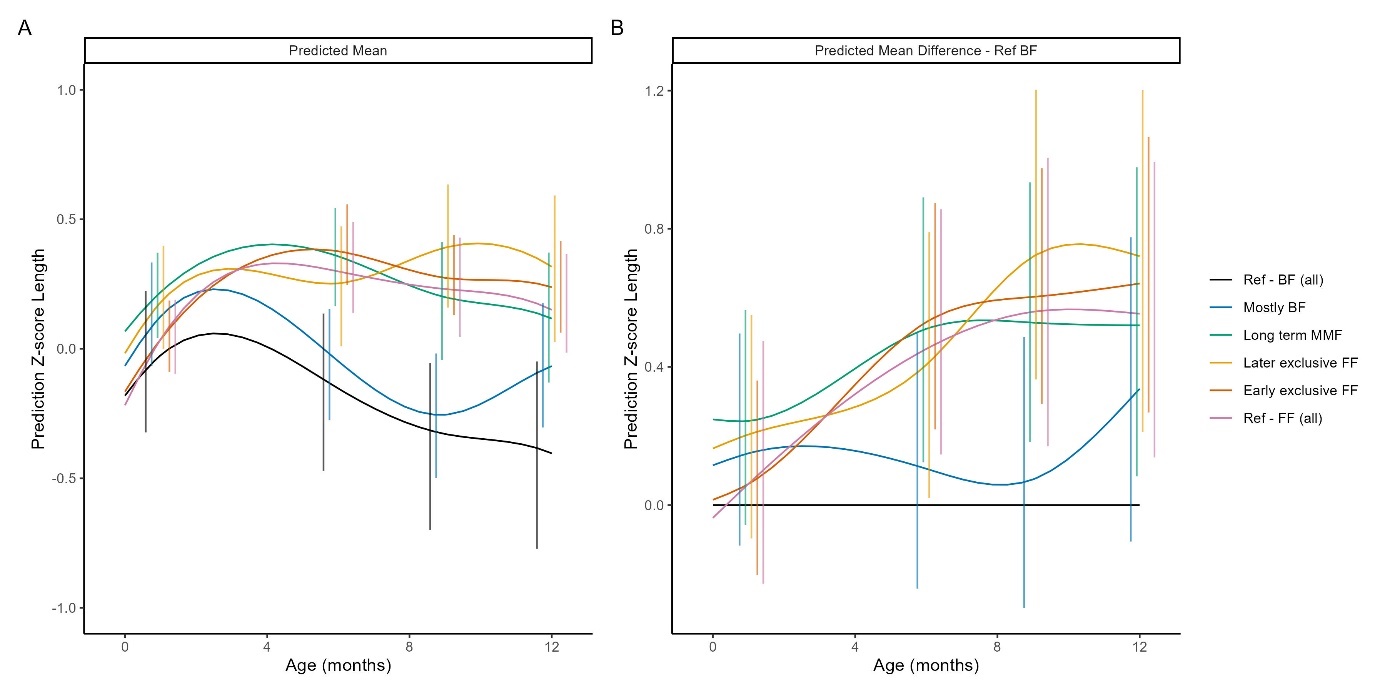


**Figure 4:** Predicted mean z-length (A) and predicted mean difference in length-for-age z-score compared to the Breastfed reference (“Ref-BF”) group (B) for the model including the main effect of feeding cluster. The model also included main effects of ethnicity, birth length, birth weight and sex, as well as their interactions with both linear and quadratic age. Error bars represent 95% confidence intervals at selected timepoints.

^Abbreviations: BF: breastfed; FF: formula feeding; MMF: mixed milk feeding; Ref: reference^

*BMI and WHO BMI-for-age z-scores by feeding cluster*


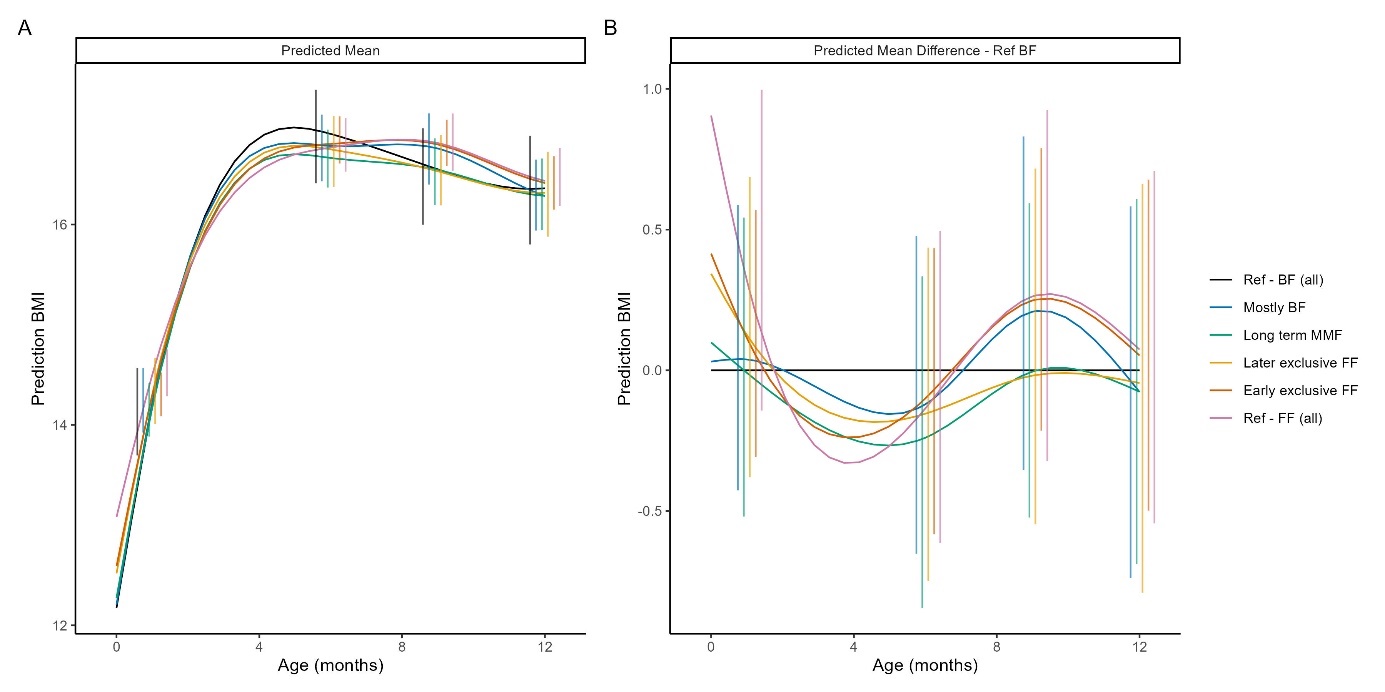


**Figure 5:** Model-predicted mean BMI (A) and predicted mean difference in BMI compared to the Breastfed reference (Ref-BF) cluster (B) for the model including the main effect of feeding cluster. The model also included main effects of ethnicity, birth length, birth weight and sex, as well as their interactions with both linear and quadratic age. Error bars represent 95% confidence intervals at selected timepoints

^Abbreviations: BF: breastfed; FF: formula feeding; MMF: mixed milk feeding; Ref: reference^

**
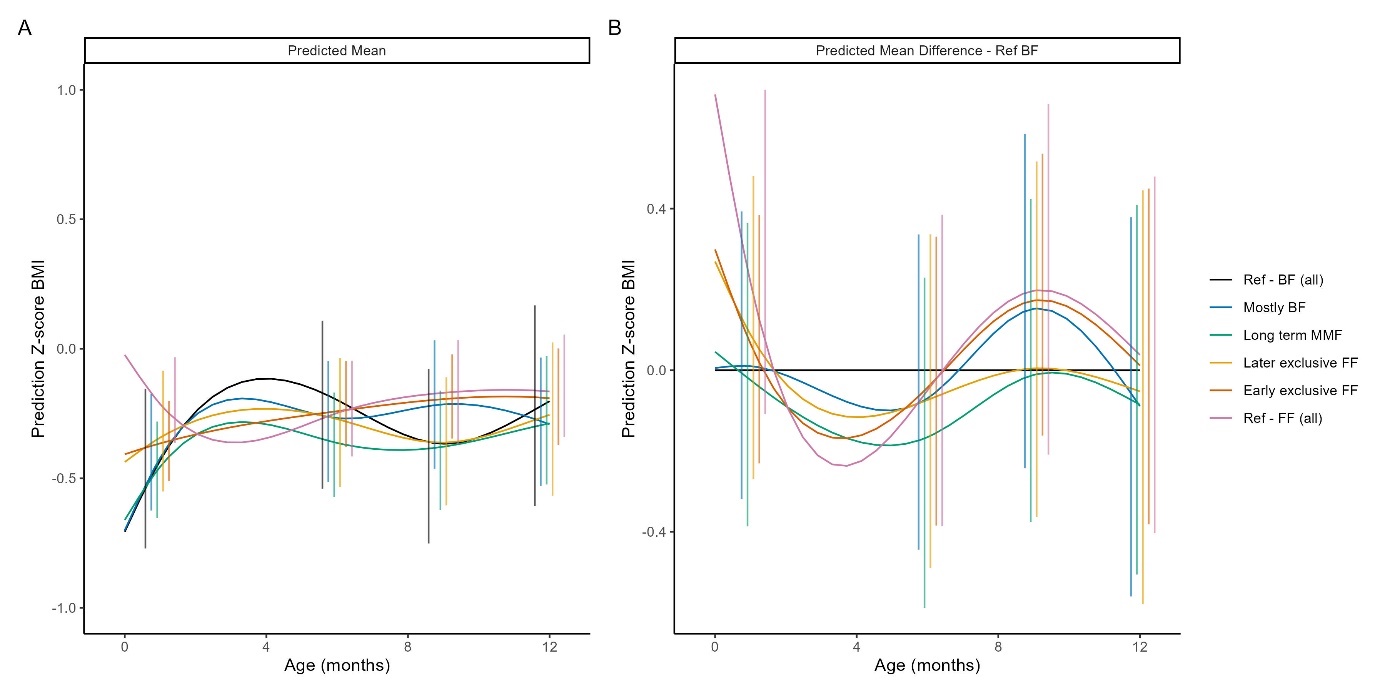
**

**Figure 6:** Predicted mean WHO BMI z-score (A) and predicted mean difference in BMI-for-age z-score compared to the breastfed reference (Ref-BF) cluster (B) for the model including the main effect of feeding cluster. The model also included main effects of ethnicity, birth length, birth weight and sex, as well as their interactions with both linear and quadratic age. Error bars represent 95% confidence intervals at selected timepoints

^Abbreviations: BF: breastfed; FF: formula feeding; MMF: mixed milk feeding; Ref: reference^

**APPENDIX 2**

**Variation in growth trajectories explained by the clustering approach**

Table 1 shows the test-statistics and p-values from comparing a full mixed-effects model including feeding cluster main effect and interactions against a null model excluding all feeding cluster terms. It can be seen that adding the feeding cluster terms in the model for weight, length, weight z-score, and length z-score significantly improved the model fit (p<0.001).

Table 1: Test statistics and p-values from comparing a full mixed-effects model including feeding cluster main effect and interactions against a null model excluding all feeding cluster terms

| **Outcome** | **Test Statistic (Chisq)** | **Df** | **p-value** | **Outcome** | **Test Statistic (Chisq)** | **Df** | **p-value** |
| --- | --- | --- | --- | --- | --- | --- | --- |
| Weight | 62.143 | 25 | **<0.001** | z-Weight | 66.887 | 25 | **<0.001** |
| Length | 61.329 | 25 | **<0.001** | z-Length | 62.581 | 25 | **<0.001** |
| BMI | 33.474 | 25 | 0.120 | z-BMI | 37.119 | 25 | 0.056 |
